# Non-Invasive Raman and XRF Study of Mīnā’ī Decoration, the First Sophisticated Painted Enamels

**DOI:** 10.3390/ma18030575

**Published:** 2025-01-27

**Authors:** Philippe Colomban, Gulsu Simsek Franci, Anh-Tu Ngo, Xavier Gallet

**Affiliations:** 1 MONARIS UMR8233, Sorbonne Université, CNRS, Campus Pierre-et-Marie Curie, 4 Place Jussieu, 75005 Paris, France; anh-tu.ngo@sorbonne-universite.fr; 2 Department of Metallurgical and Materials Engineering, Faculty of Engineering, Istanbul Gedik University, Cumhuriyet Mah. İlkbahar Sok. No: 1 Kartal, 34876 Istanbul, Türkiye; gulsu.simsek@gedik.edu.tr; 3 UMR 7194—Histoire Naturelle de l’Homme Préhistorique (HNHP), Musée National d’Histoire Naturelle, CNRS, Université Perpignan Via Domitia, Musée de l’Homme, 17 Place du Trocadéro, 75116 Paris, France; xavier.gallet@mnhn.fr

**Keywords:** Iran, Seljuks, mīnā’ī, painted enamels, composition, pigments, Raman spectroscopy, pXRF, thickness, İznik, blue, white, fritware, boron, cassiterite

## Abstract

Mīnā’ī wares, crafted during the 12th–13th centuries, represent some of the earliest examples of sophisticated painted enamel decoration by potters. Due to the thinness of these enamel layers, their detailed characterization remains challenging, even with the use of advanced techniques, such as Proton-Induced X-ray Emission (PIXE) analysis and Rutherford Backscattering Spectrometry (RBS). This study provides the first combined non-invasive analysis, using X-ray fluorescence (XRF) and Raman spectroscopy, of five shards attributed to mīnā’ī wares. For comparison, two İznik shards from the 17th century, which feature similarly styled but thicker enamel decorations, were also analyzed. Interestingly, the mīnā’ī paste was found to contain lead and tin, suggesting the use of a lead-rich frit in its composition. This finding was confirmed through micro-destructive analysis, using Scanning Electron Microscopy with Energy Dispersive Spectroscopy (SEM–EDS). Elements, such as rubidium (Rb), strontium (Sr), yttrium (Y), and zirconium (Zr), produced significant XRF signals and effectively distinguished mīnā’ī wares from İznik wares. A uniform tin-rich glaze, measuring 300–500 µm in thickness, was used as a base layer for the much thinner painted mīnā’ī enamels. The colored areas (blue, turquoise, red, green, black, white, eggplant) revealed the presence of various coloring agents and phases, such as spinels, chromite, and ions like Cu^2+^ and Co^2+^, as well as opacifiers like cassiterite and lead–calcium/potassium arsenates. Two distinct cobalt sources were identified: one associated with arsenic and the other with manganese and nickel. These cobalt sources are comparable to those used in İznik pottery. For the first time, boron was detected in the blue enamel of mīnā’ī wares.

## 1. Introduction

Mīnā’ī wares, produced during the 12th and 13th centuries, are regarded as the first sophisticated painted enamel decorations, created by potters. These ceramics are considered among the most luxurious in the Islamic world [1,2,3,4,5,6]. According to McClary [7] and François [8], some argue that no evidence exists of mīnā’ī production before 1180 [5]. However, the Konya palace kiosk (the ancient capital of the Seljuk Sultanate, Türkiye), securely dated to the 1170s, was decorated with numerous mīnā’ī tiles. Production flourished in Iran until the early 13th century, ceasing with the Mongol invasion. Cities involved in the production of mīnā’ī wares are believed to include Kashan, Sava, Rayy, and Natanz [9,10].

The earliest Persian miniature manuscripts, dating back to the 12th century [11,12,13], display clear parallels with Seljuk mīnā’ī wares in terms of inspiration and technique. Similarities with Chinese art are also evident in both pottery and manuscript decoration [10]. All mīnā’ī wares are thought to have been produced on stonepaste (fritware) bodies [2,3,4,5,6,7,8,9,10]. A well-documented recipe for stonepaste is found in a treatise written in 1301 (700 Hegira) by Kāshānī potter Abū’l-Qāsim, translated by Allan [14]. The recipe includes 10 parts quartz, called “sugary stone” (*shukar-i sang*), ground and sieved through coarse silk (corresponding to a grain size of 1–10 µm), combined with one-part ground glass frit and one-part white clay. Holakooei [15] recently provided additional details on this process.

While most mīnā’ī wares are decorated over a white glaze, rare examples with turquoise-glazed bases exist. These, however, are limited and remain analytically understudied, particularly regarding their painted decorations. The most comprehensive study to date, employing Rutherford Backscattering Spectrometry (RBS) and Proton-Induced X-ray Emission (PIXE) analysis, was conducted by Nikbakht and Montazerzohouri in 2021 [16]. They highlighted the exceptional thinness of painted enamels, often just a few microns thick, applied over a glazed background. Their composition varied according to color: the PbO content ranged from 2 to 3 wt% for dark blue and black, up to 10 wt% for turquoise, and more than 30 wt% for cream, red, and some blues. The SnO_2_ content ranged from 4 to 10 wt% in cream and red, but was absent in other colors. Notably, the authors acknowledged that these results include contributions from the deeper substrate, complicating the reliability of the analysis.

The limitations of advanced techniques, such as Rutherford Backscattering Spectrometry (RBS) and Proton-Induced X-ray Emission (PIXE) analysis, when analyzing thin enamel layers motivated us to explore more accessible and increasingly portable methods [17], specifically portable X-ray fluorescence (pXRF) and Raman microspectroscopy.

While XRF analysis shares some of the same uncertainties as PIXE analysis, particularly regarding the depth of analysis in materials with layers of varying and often unknown thickness, Raman analysis offers greater control over the analyzed volume. By adjusting the illumination and light-collection optics, Raman microspectroscopy can precisely target volumes ranging from cubic micrometers (µm^3^) to several cubic millimeters (mm^3^), with even broader areas accessible through the use of mapping techniques.

Recent preliminary non-invasive Raman spectroscopy studies on Persian pottery confirmed the variability in the glaze compositions of a mīnā’ī bowl depicting *Faridun astride the cow*, *Birmaya*, dated to the mid-12th to early 13th centuries. Interestingly, cassiterite was present only in the turquoise and green areas [17]. The present study is the first comprehensive non-invasive analysis of seven shards using both Raman microscopy and XRF analysis.

The accuracy of certain attributions requires further evaluation. Two of the examined samples, which share similar motifs as expected in mīnā’ī wares, are believed to have been produced during a later period (i.e., post-16th century), by the Ottoman-era İznik tile kiln workshops. A comparison will involve analyzing the glaze signatures using Raman spectroscopy, alongside examining the chemical compositions of the glaze and pigments with XRF analysis. Previous studies have reported the Raman spectra [18,19] of certain colored areas and have provided body composition details of other samples [10,15,20,21].

## 2. Materials and Methods

### 2.1. Artefacts

**Figure 1:**
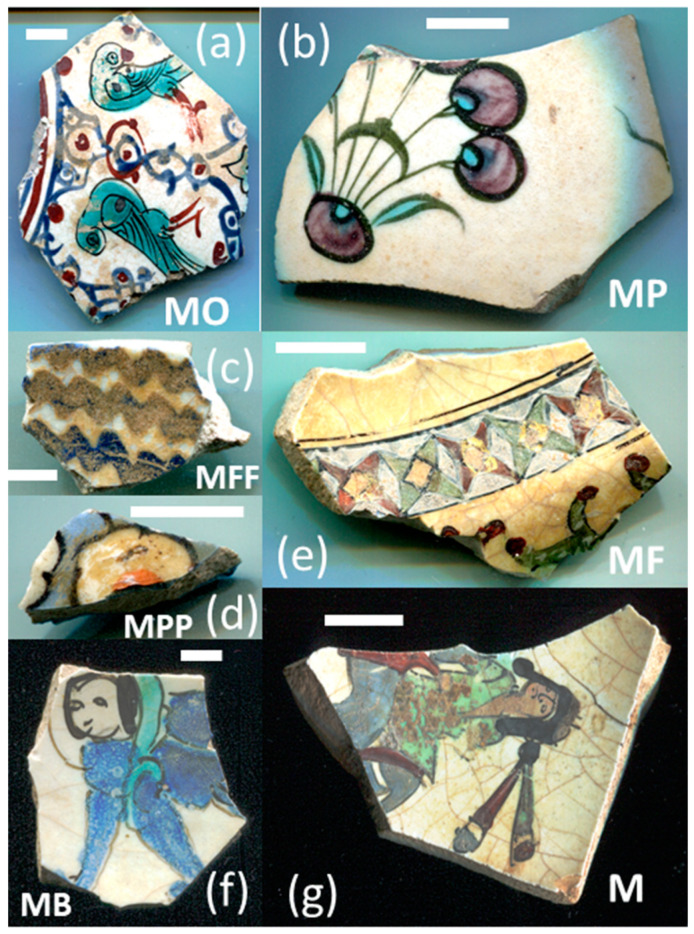
Studied samples decorated with: (**a**) two birds (MO, mīnā’ī, ~7 × 5.5 × 0.45 cm^3^), (**b**) peacock feathers (MP, İznik, ~7 × 4 × 0.3 cm^3^), (**c**) a zig-zag black frieze (MFF, mīnā’ī, ~4 × 3 × 0.5 cm^2^), and (**d**) a flower (MPP, İznik, ~2 × 1 × 0.55 cm^3^), (**e**) a frieze of stars (MF, mīnā’ī, ~5 × 4 × 0.5 cm^3^), (**f**) a blue sphinx (MB, mīnā’ī, ~4 × 3 × 0.4 cm^3^), and (**g**) a horseman figure (M, mīnā’ī, ~7.5 × 5 × 0.5 cm^3^). Scale bar: 1 cm.

Figure 1 illustrates the decorated surfaces of shards from Soustiel’s collection. A portion of this collection has been the subject of previously published works [2,22]. The shards feature motifs resembling the horseman and sphinx decorations found in the British Museum (inv. 1930.0719.63) and the Metropolitan Museum of Art in New York [7]. In the 12th and 13th centuries, the sphinx was strongly associated with light and was regarded as a celestial creature of paradise [7,23]. Star motifs were also common, often appearing as star-shaped tiles [1,2,3,7]. Birds and peacock feathers frequently appeared in Persian poetry and miniatures [12,23,24]. However, peacock feather decorations (MP) are also associated with Damascus-style production in İznik [25]. The thickness of the black contours suggests a later production period, specifically the 17th century. The flower motif with a red-colored center (MPP) is a distinctive feature of İznik pottery, likely created by an İznik potter [2,26]. Friezes were popular decorative patterns [12], with many motifs traced back to the Sassanid era (3rd–7th centuries). Comparative analyses of these motifs are discussed in references [24,27,28,29].

### 2.2. Techniques

#### 2.2.1. Optical Microscopy

Sections and surfaces of each shard were observed without any preparation, using a BX51 Olympus microscope, equipped with 5× (NA: 0.15) and 10× (NA: 0.30) objectives, and AnalySIS software (Olympus Soft Imaging Solutions, Tokyo, Japan).

#### 2.2.2. Scanning Electron Microscopy–Electron Dispersive Spectroscopy

After the XRF and Raman analyses, small grains (less than 2 × 2 × 2 mm^3^) were taken from the shard’s corners for additional SEM–EDS analysis, using a JEOL 5410LV SEM–EDX (Tokyo, Japan), with a 20 kV acceleration voltage. The samples were partially wrapped with carbon-rich conductive tape to minimize charging effects, leaving a small, exposed window for analysis. This method, while less effective than applying a conductive coating (C or Au–Pd), preserves the sample. The exposed window facilitates the correlation of Raman and SEM–EDS measurements, as recognizing specific colored areas on SEM images can be challenging. The EDS spectra were recorded at ×400 magnification, over a sub-millimeter area (~300 × 250 µm^2^), much larger than the grain size, but these measurements cannot be considered average values. The uncertainty of the measurements is less than 5 at.% for the major elements and 10 at.% for the minor ones.

#### 2.2.3. Portable X-Ray Fluorescence Spectroscopy (pXRF)

The procedure followed is detailed in previous studies [30,31]. X-ray fluorescence (XRF) analysis was performed using a portable instrument (Elio, Milano, Italy), equipped with a miniature X-ray tube system, featuring a Rh anode, a ~1 mm^2^ collimator, and a large-area Silicon Drift Detector, with an energy resolution of <140 eV for Mn Kα. The instrument detected an energy range from 1.3 keV (in air) to 43 keV. The instrument was positioned on a dedicated frame support and the measurements were conducted in point mode, with a 180 s acquisition time at 50 kV and 80 μA, with no filter between the tube and the sample. The analysis depth, estimated using the Beer–Lambert law, is defined here as the thickness of the top layer from which 90% of the fluorescence originates [32]. This depth is approximately 6 μm for Si Kα, 170 μm for Cu Kα, 300 μm for Au Lα, and 3 mm for Sn Kα. These values should be compared with the thicknesses of the painted enamels, which range from a few microns to several tens of microns [16,17,21].

Accuracy was checked by measuring the reference glass and stone samples [30,31].

The intensities of the peaks in the electronic transitions (Kα, Kβ, Mα, Mβ, Lα, Lβ, Lγ, etc.), giving the characteristic peaks, depend firstly on the elements concerned and secondly on the contents of these elements. This is why, for example, the peak intensity of the major element Si (silicon) can be smaller than those of minor elements (e.g., Pb) and comparable to that of Rb traces. In addition, a complex-colored decoration requires a topological variation in the three dimensions of the coloring agent concentration. The calculus of a “composition”, therefore, has no meaning, and this is why we developed the clustering analysis procedure in different diagrams, comparing the areas of the characteristic peaks. This procedure, acquired during the analysis of hundreds of objects using Artax 7.4.0.0 (Bruker, AXS GmbH, Karlsruhe, Germany) software, is described in previous papers [30,31]. The net area under the peak at the characteristic energy of each element, selected from the periodic table, was calculated and the area of the major, minor, and trace elements were determined in the colored areas. Before plotting the diagrams, the net areas of each element were normalized using the number of XRF photons derived from the peak in the X-ray tube rhodium anode. In some cases, normalization with respect to the signal of another element (e.g., Co for a blue area) was also made for comparison. Then, the data were plotted in ternary or biplot scattering diagrams. Additionally, Euclidian hierarchical similarity plots with average linkage (Ward’s distance) were drawn for the interpretation and discussion of the results, using Python software (version 3.12.4).

#### 2.2.4. Raman Microspectroscopy

The shards were analyzed in a laboratory using a Labram HR800 spectrometer (HORIBA Scientific Jobin-Yvon, Palaiseau, France), equipped with an Ar^+^ ion plasma laser, Innova I90C 6UV (Coherent Inc., Santa Clara, CA, USA).

The 457.9 and 514.5 nm lines of the Ar^+^ laser were employed in the laboratory, with the illumination power at the sample surface ranging from approximately 0.3 mW (for colored enamels) to 8 mW (for paste) for the blue line and from 0.2 mW to 2mW for the green line. A comparison of the spectra facilitated the separation of vibrational and luminescence features. A long-working-distance (LWD: 13 mm) 50× (NA: 0.45) microscope objective (Olympus Corp., Tokyo, Japan) was used for the analysis. The spot sizes were approximately 5 × 5 µm^2^, closely matching the expected pigment grain size. In-depth laser penetration was similar for colorless glaze, but significantly reduced for dark-colored areas. The spectral data were recorded across a window from 50 to 4000 cm^−1^. Counting times ranged from a few minutes to several tenths of a minute, with at least three accumulations performed to suppress cosmic ray signals. For each area considered, three spots were systematically analyzed. Using a 600 lines/mm grating, the uncertainty in the peak wavenumber was less than ±2 cm^−1^.

## 3. Results

### 3.1. Microstructures

Figure 1 illustrates that most of the shards exhibit a crack network, indicative of poor compatibility between the thermal expansion coefficients of the glaze and the body. This suggests that the glaze was not optimized for the quartz-rich body, possibly due to the use of glazes initially developed for other substrates. Visual examination led to the assignment of the MP and MPP shards to İznik kilns, the other shards being mīnā’ī wares.

**Figure 2:**
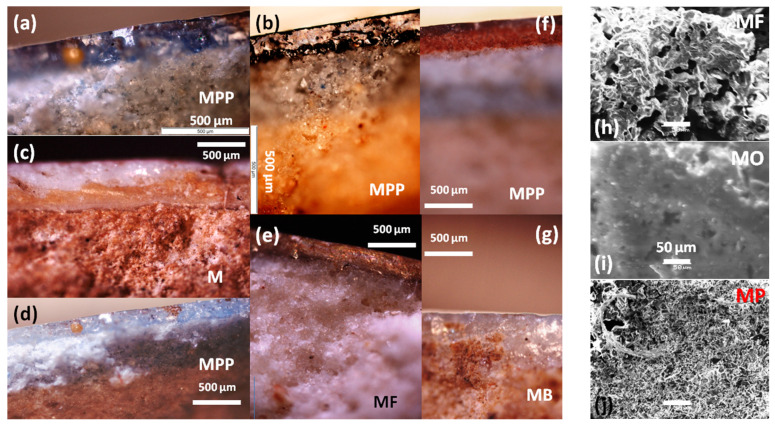
Selected optical views of shard sections (without polishing to preserve the samples): (**a**,**b**,**d**) MPP blue background glaze (İznik), (**c**) M horseman brown saddle glaze (mīnā’ī), (**e**) MF frieze section with yellow background glaze (mīnā’ī), (**f**) MPP red enamel on blue background (İznik), and (**g**) MB sphinx blue background (mīnā’ī). SEM views of (**h**) MF, (**i**) MO, and (**j**) MP body fractures.

**Figure 3:**
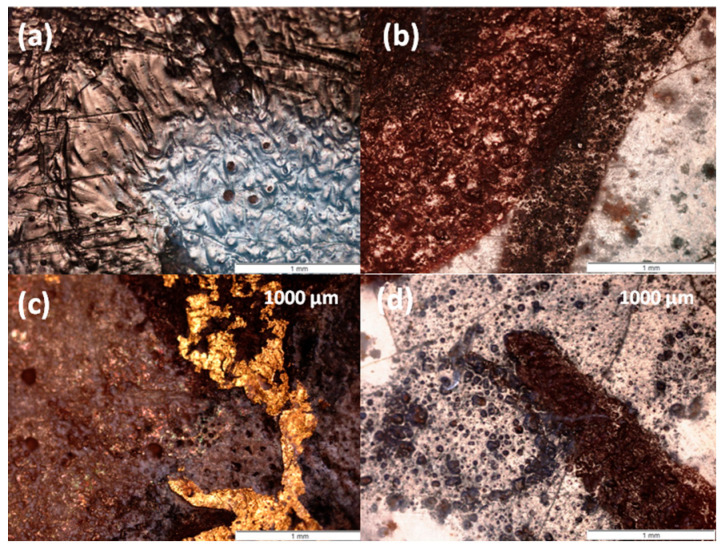
Selected optical surface views: (**a**) green spot in the MP peacock feather (İznik), (**b**) red and black line on the MO bird couple mīnā’ī shard, (**c**) gold foils on the MF frieze mīnā’ī shard, and (**d**) black line on blue enamel on the MO bird couple mīnā’ī shard.

Figure 2 presents photomicrographs of shard cross-sections. Some shards have a white paste (e.g., MF and MP), while others exhibit a pinkish tone. The fractures in most of the shards resemble that of a sugar cube (Figure 2e,h,j), typical of fritware, where grains of sand are lightly cemented by intergranular glass. The mean grain size varies between ~2 µm (MP, Figure 2j) and 10 µm (MF, Figure 2h). In contrast, the microstructure of the MO shard appears denser and more glassy. The thickness of the base glaze layer, white or cream (M, MB, MF, MO, and MP) and turquoise–blue (MPP), ranges from 200 to 500 µm. The painted enamels are much thinner, approximately 100 µm for the red layer in photo 2f (MPP), with evidence of glaze diffusion into the paste. The black lines are even thinner, as seen in the surface views (Figure 3). A white background layer is clearly visible between the paste and the glaze in the MPP shard (Figure 2a,d,f), consistent with production in İznik [2,17,25].

The painted enamel appears completely melted, as evidenced by the presence of bubbles (Figure 3a) and segregation into islands, resembling oil drops on the surface of water (Figure 3d). This suggests the use of a highly volatile flux, such as a boron-rich composition derived from borax, which forms very fluid phases when combined with lead oxide. The gilding is also thin, typically around one micron, as is standard practice. In contrast, the surface of the MP shard (İznik) displays a wrinkled and bubbled appearance, due to its significantly greater thickness.

### 3.2. Information on Elemental Composition

**Figure 4:**
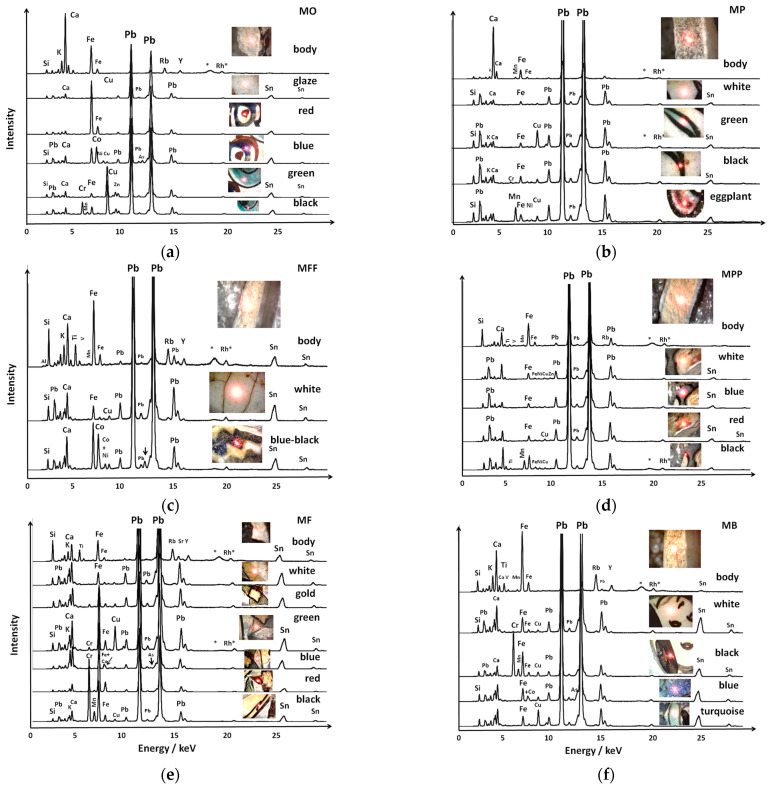
Selected XRF spectra of: (**a**) two birds (MO, mīnā’ī), (**b**) peacock feathers (MP, İznik), (**c**) a zig-zag black frieze (MFF, mīnā’ī), (**d**) a flower (MPP, İznik), (**e**) a frieze of stars (MF, mīnā’ī), and (**f**) a blue sphinx (MB, mīnā’ī). The spectra of lead-rich silicates have been amplified, by a factor of 5 to 8, to enhance the visibility of the peaks of other elements. Each analyzed area is visually represented, with the red spot marking the laser focus adjustment point. Peaks labeled with * and Rh* correspond to Compton scattering and the characteristic X-ray emission from the instrument anode, respectively.

**Figure 5:**
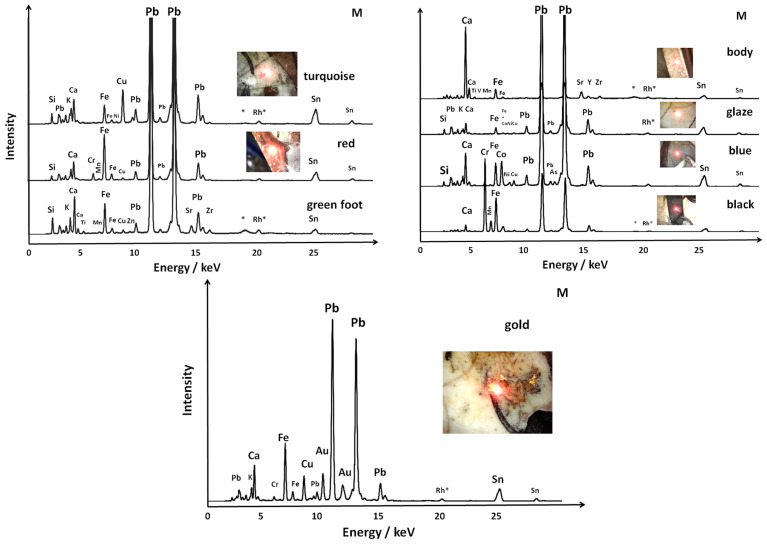
Selected XRF spectra recorded from different areas of the mīnā’ī shard depicting a horseman (M). Some spectra of lead-rich silicates have been amplified, by a factor of 5 to 8, to highlight the peaks of other elements. The Compton (*) and anode emission (Rh) signals are labeled.

**Figure 6:**
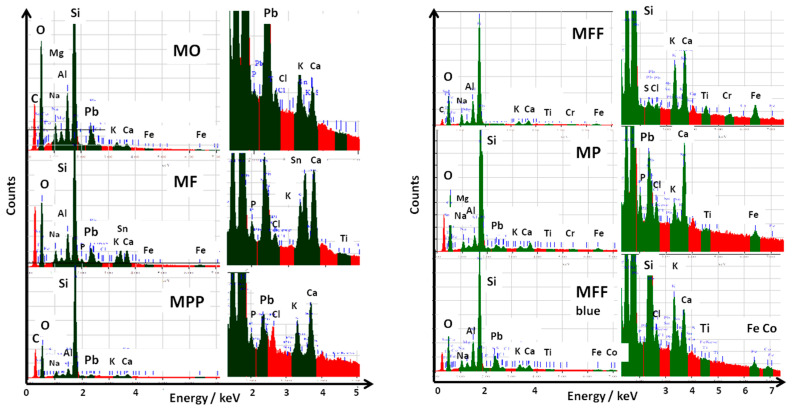
Selected SEM–EDS spectra from the paste (fractures of MO and MF mīnā’ī shards and MPP and MP İznik shards) and the blue area of the MFF shard. Zoomed-in views of the 1 to 5 keV spectral range are shown on the right side. The signal used in the calculation is shown in green.

**Figure 7:**
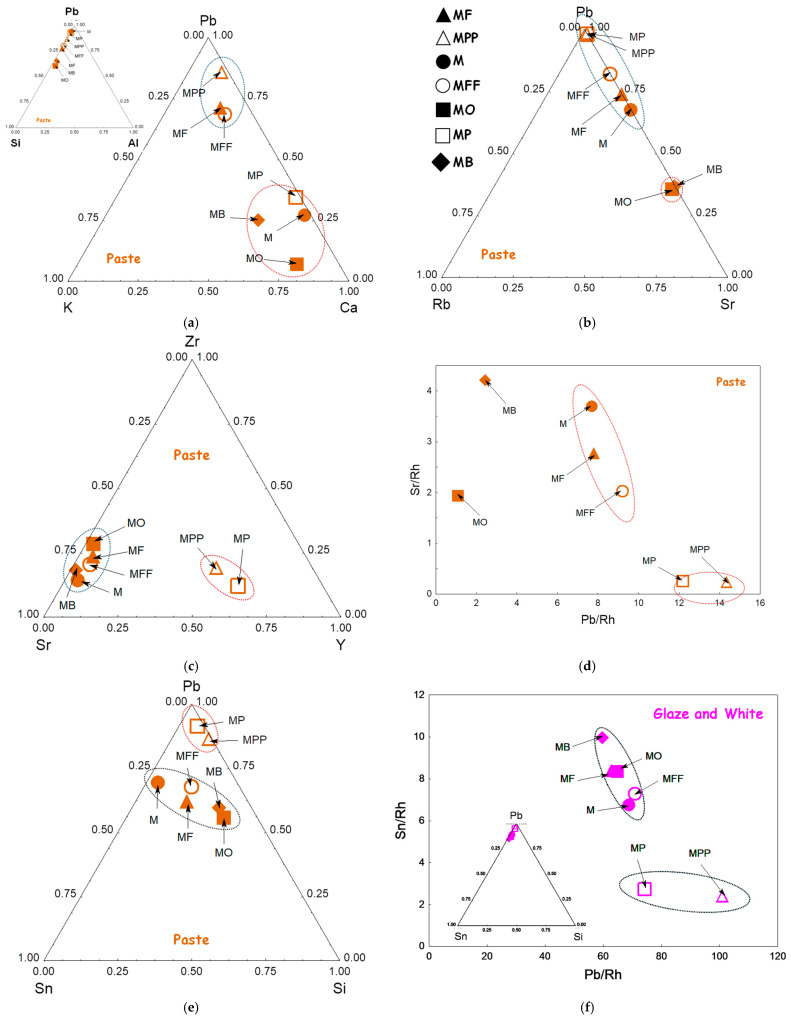
Comparison of the peak area for Pb, K, and Ca flux elements (**a**); Pb and Rb and Sr impurities (**b**); Zr, Sr, and Y impurities (**c**); as well as Sn vs. Pb in the body (**e**); Sr vs. Pb (**d**) and Sn vs. Pb (**f**), normalized with Rh signal are given for the paste and the glaze, respectively.

Selected XRF spectra are shown in Figure 4 and Figure 5. The SEM–EDS spectra are shown in Figure 6 and Figure 7.

#### 3.2.1. Paste Composition

Visual examination of the body spectra reveals differences in the relative intensities of the peaks for elements such as Ca, Fe, Si, Sn, and Pb. The variation in the intensity ratios of the Fe Kα/Si Kα peaks is consistent with the two types of paste coloring: white (MF and MP) and pinkish (others). The calcium peak intensities are higher in the MO, MP, and MB shards, while they are minimal in the MPP and MF shards. Unexpectedly, some paste spectra show trace amounts of lead and tin, particularly in the M, MF, and MFF mīnā’ī shards. Traces of lead and tin are also detected in the MO and MB mīnā’ī shards. The pastes of the MP and MPP shards appear almost free of tin, consistent with some İznik production [33].

The EDS spectra (Figure 6) and the calculated compositions (Table 1) confirm the results of the XRF analyses. The pastes are highly siliceous, particularly in İznik shards, with sodium being the main source of flux. Small quantities of lead and tin are detected. The SEM–EDS measurements taken at a much smaller scale (~7.5 × 10^−2^ mm^2^) than the XRF analysis show the heterogeneous distribution of tin and lead. The mean composition of mīnā’ī paste, calculated from the data in Table 1, is approximately at % Si 25.3, Al 3.5, Mg 0.77, Na 3.39, K 0.77, Ca 0.92, Pb 1.17, and Sn 0.36. This corresponds to the following wt% oxide composition: wt% 62 SiO_2_—14.5, Al_2_O_3_—1, MgO—5.5, Na_2_O—3, K_2_O—2, CaO—10.5, and PbO—2 SnO_2_. These values are in good agreement with previous data [16,33], except for the significantly higher aluminum content. İznik production from the 16th and 17th centuries is characterized by high quartz content (65–85% SiO_2_) and low alumina levels (3–6 wt% Al_2_O_3_), reflecting the typical fritware/stonepaste recipe commonly used in the production of “İznik” tiles [33]. The findings shown in Table 1 confirm that the MP and MPP shards belong to the İznik-production group.

The decision to avoid quartz grains in the EDS measurement likely contributed to the elevated aluminum levels observed. Indeed, considerable compositional heterogeneity (a factor > 2 for some elements) is observed within the same sample. The iron content is low and the high amount of silica in the İznik shards is highlighted by the large number of quartz grains.

The presence of tin does not result from contamination during the firing of lead-based enamels, as the measurements were made at the breakage of the shards, most of which were far away from the surface. Moreover, tin was detected in most of the spots. It is plausible that the tin originates from the lead source used, likely waste from bronze manufacturing, as Persian bronzes were made from copper–tin alloys containing approximately 20% lead [34]. To our knowledge, the presence of tin in mīnā’ī paste, undoubtedly sourced from lead-rich frit glass, has not been highlighted previously [35]. The Pb/Sn ratio ranges from 0.5 to 50, with the central distribution being between 4 and 10, which is consistent with the ratio of 5 observed in Persian bronze [34]. The inability to polish small fragments (~1 to 5 mm^3^) from certain shards means that the proportion of the vitreous phase and its distribution between quartz grains cannot be fully observed. The area analyzed is relatively large compared to the grain sizes (~300 × 250 µm^2^ versus ~1 to 20 µm in grain diameter), but significant compositional heterogeneities are observed (as seen in the different measurements for the same shard). For example, the calcium content varies by a factor of 3 (MF mīnā’ī) or 4 (MPP İznik), while the sodium content varies by a factor of 2 (MPP İznik). The MP1 shard stands out for its lower flux content, which is compensated by a much finer grain size, facilitating sintering. Aside from the higher silica content, similarities between Ottoman and mīnā’ī pastes are also noted.

The measurements taken near the glaze reveal small amounts of cobalt and chromium, likely due to the intrusion of the enamel as it melts into the cracks and the porosity of the paste. This behavior is consistent with the low viscosity of the enamel at firing temperature and the thinness of the painted decoration.

The significant presence of phosphorus in some of the shards appears to be too high (up to 2.5 at %, with mean values of ~0.5 and 0.15 for İznik and mīnā’ī paste, respectively) to be attributed solely to contamination from prolonged exposure to phosphorus in the ground (since the objects come from excavations). The presence of phosphorus suggests the use of raw materials containing phosphate. The incorporation of bone into glass production was practiced by Sasanian glassmakers [36] and these glasses also contain small amounts of sulfur and chlorine. Such glass may have been used to prepare the frit used for cementing sand.

#### 3.2.2. Glaze Composition

Due to the interdiction of sampling from areas other than the corner of one İznik shard (MPP, see Appendix A, Table A2), pXRF measurements were taken from the upper surface of the shards.

### 3.3. Classification Based on Composition Ratios Measured by pXRF

**Figure 8:**
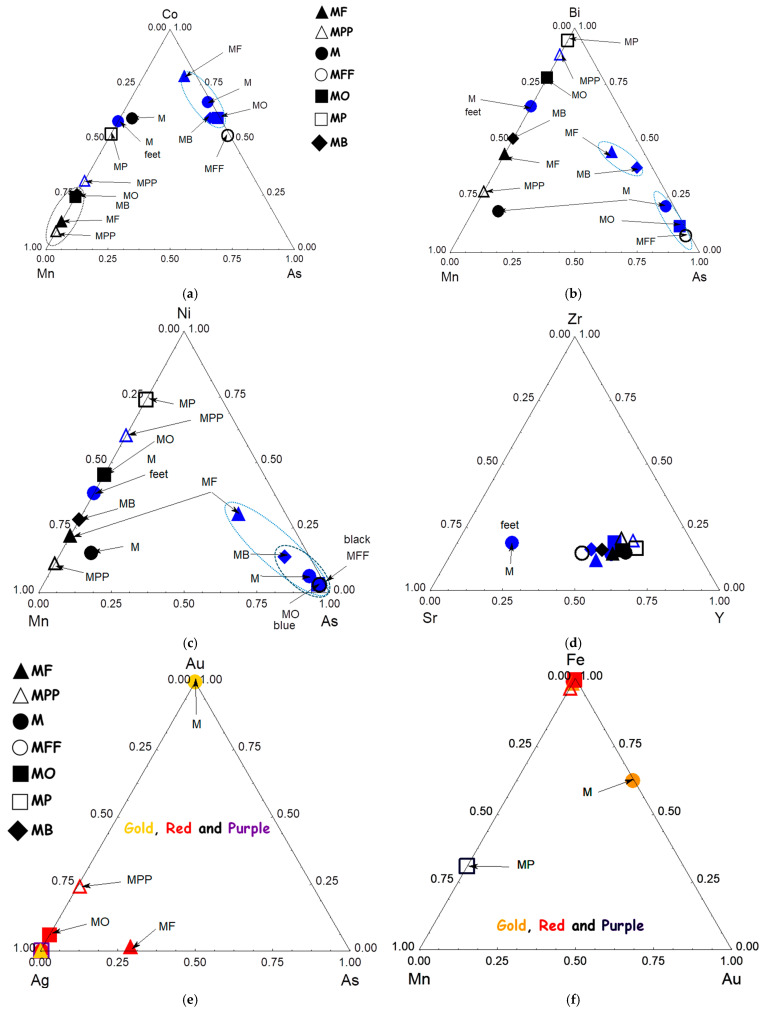
Comparison of the peak area for Co and associated elements for blue and drak blue to black areas (**a**–**c**); comparison of the Zr, Sr and Y impurities signal for glassy matrix impurities (**d**); comparison of Au, Ag and As and Au, Mn and Fe signalfor chromophore and associated elements related to red, purple, and gilding (**e**,**f**).

Figure 7 and Figure 8 compare the ternary diagrams built using the data on the areas of the peaks related to the elements with visible pXRF peaks.

#### 3.3.1. Body

The Pb–K–Ca ternary diagram visualizes the variability in the lead content, as detected by pXRF analysis (Figure 7a). The measurements reveal at least two types of paste: the MF and MFF shards are lead rich, while the MP, MB, M, and MO shards contain more calcium. This relationship between mīnā’ī and İznik pastes is confirmed. The MPP and MP shards, however, show the lowest potassium content, clearly differentiating the two types of ceramic bodies.

The examination of the signals related to impurities, such as Rb and Sr (Figure 7b), and Zr, Sr, and Y (Figure 7c) (whose XRF signals are significant even at low concentrations and, thus, serve as reliable markers), further confirms the distinctions between the groups. The levels of earth-alkaline impurities (e.g., Sr) corroborate the division of mīnā’ī shards into three groups, namely the MO and MB shards (first group), the M and MF shards (second group), and the MFF shard (third group), with the MPP and MP shards being associated with İznik production. The Zr–Sr–Y ternary diagram (Figure 7c) and the Sr vs. Pb scattering biplot support this classification, demonstrating the efficiency of pXRF in distinguishing pastes based on impurities from raw materials.

The ternary diagram based on Pb, Sn, and Si signals (Figure 7e) further supports this classification, with the mīnā’ī group (MO, M, MB, MF, and MFF) exhibiting higher tin content.

#### 3.3.2. Background Glaze

The Pb–Sn–Si diagram (Figure 7e) does not differentiate between the glazes covering the paste. However, the Sn/Rh vs. Pb/Rh scattering biplot (Figure 7f) successfully identifies the two groups already distinguished from the paste analyses, namely the MP and MPP İznik shards on one side, and the other shards, on the other side.

However, the Sr/Rh vs. Pb/Rh scattering biplot (Figure 7d) also reveals three groups within the mīnā’ī paste group: one group consists of the M, MF, and MFF shards, while the MB and MO shards are sufficiently distinct to belong to separate groups. This suggests the use of different raw materials, likely indicating different production locations or time periods. More samples are needed to verify this conclusion.

#### 3.3.3. Cobalt and Associated Elements

Figure 8 compares the peak areas characteristic of cobalt, blue chromophore, and the associated elements characteristic of primary type mineral deposits (Mn) or hydrothermal veins (As, Bi, Figure 8a,b). Nickel is common to both types of geological contexts and is less efficient (Figure 8c) to classify than cobalt sources [37,38]. Two groups are clearly identifiable, on the one hand, there are the MF, M, MO, and MB mīnā’ī shards, which use cobalt associated with arsenic, and, on the other hand, there is the MPP İznik shard, whose cobalt is associated with manganese and nickel, like the bluish flow on the foot of the M shard (probably the result of contamination during firing in the kiln). This colored area also contains bismuth (Figure 8b). The Bi–Mn–As ternary scattering diagram classifies two groups very well: the M, MB, and MF shards on one hand, and the MO, M, and MFF shards, on the other.

The black-colored areas are based on manganese, but also contain some cobalt, bismuth, and nickel.

Ores collected from the site of some ancient Iranian mines [39] were studied by Matin and Pollard [40,41]. The ores contain arsenic, which is compatible with the measurements here, but nickel and bismuth were not measured by Matin and Pollard.

#### 3.3.4. Other Colors and Luster

The impurity diagrams (Figure 8e,f) do not provide any clear discrimination criteria. Traces of silver are detected in the red areas of the MO, M, and MF shards, which may indicate that some parts underwent a luster-type preparation [42] that has since degraded over time. Luster has been reported in mīnā’ī wares [2,16].

Gilding was applied using pure gold for the M shard, but with an alloy of gold and silver for the MF shard (Figure 8e).

The red color is derived from iron, while the purple/eggplant color is attributed to manganese (Figure 8f).

### 3.4. Phase Characterization

**Figure 9:**
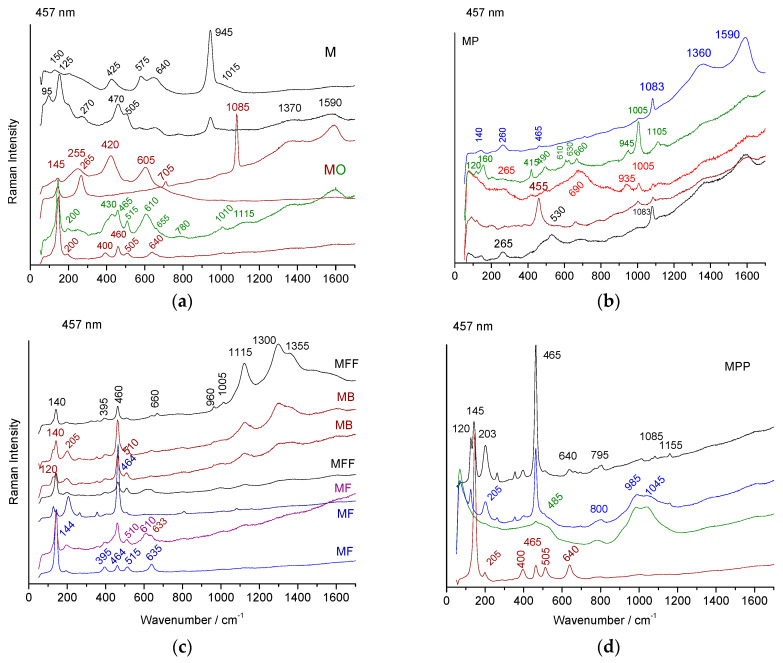
Selected Raman spectra, recorded using blue laser excitation, for different points in the body facture section of M (**a**), MO (**a**), MP (**b**), MB (**c**), MF (**c**), MFF (**c**), and MPP (**d**) shards.

**Figure 10:**
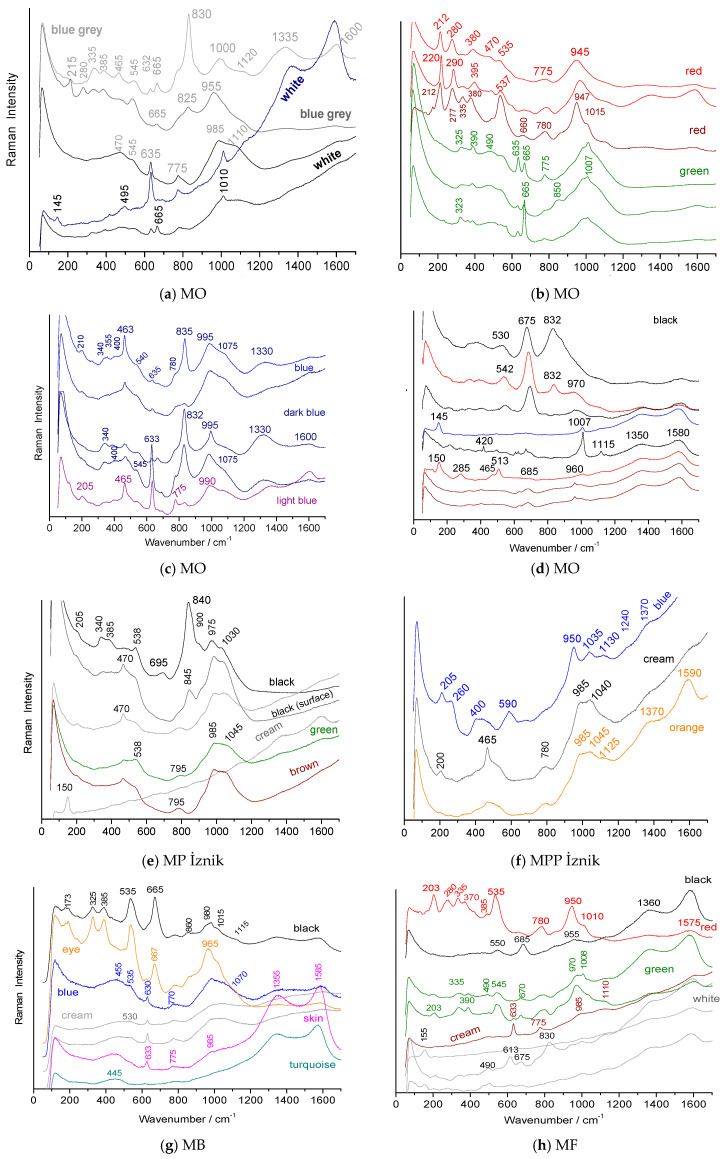
Selected Raman spectra, recorded using a blue laser, for various points on the shard surfaces of MO (**a**–**d**), MP (**e**), MPP (**f**), MB (**g**), and MF (**h**).

**Figure 11:**
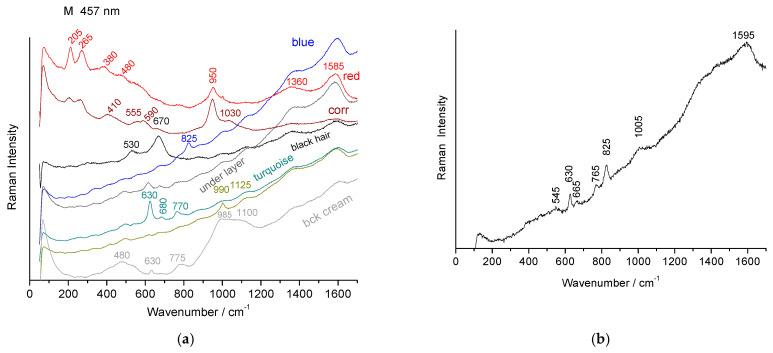
Selected Raman spectra, recorded with a blue laser, for different points of the shard surface of the M (**a**,**b**) shard (**b**, face).

Simple elemental analysis does not make it possible to identify in what form the coloring element is found. Selected Raman spectra, recorded based on different spots on the shards, are shown in Figure 9, Figure 10 and Figure 11.

#### 3.4.1. Raman Signatures of the Body

The Raman spectra firstly show the very characteristic spectrum of quartz, with a stronger ~465 cm^−1^ peak (bending SiO_4_ mode), a broad librational mode at ~200 cm^−1^, a lattice mode at 100 cm^−1^, and series of narrow small peaks between 200 and 1155 cm^−1^, as previously observed [43] (see examples of nearly ‘pure’ quartz spectrum in Figure 9c,d). Additionally, the spectra characteristic of titanium oxide, rutile (broad bands at 255, 420, and 605 cm^−1^, Figure 9a), and anatase (a strong narrow peak at 144 cm^−1^ and a broader peak at 400 and 505 cm^−1^ [44]) are observed, according to the detection of titanium using SEM–EDS (Table 1). Due to the huge Raman intensity of these phases, these Raman signatures are observed in many pieces of pottery [45]. Calcite, with its characteristic narrow 1085 cm^−1^ peak [46], is detected in the M (Figure 9a) and MP (Figure 9b) samples, according to the XRF measurements (Figure 5 and Figure 4). In the latter shard, gypsum is observed in the body of the shard (a narrow peak at ~1005 cm^−1^ and smaller peaks at 180, 412, 490, 610, and 1130 cm^−1^ [47], Figure 9b), according to the detection of sulfur using EDS (Table 1, Appendix A). The strong peak at 945 cm^−1^ (Figure 9a,b) might correspond to a phosphate [48,49], phosphorus being detected by the SEM–EDS (Table A1, Appendix A).

The spectra characteristic of a glassy phase are recorded for some spots (Figure 9b). The center of gravity of the stretching band of the SiO_4_ tetrahedron, the main vibrational unit of silicate [50], ranges between 985 and 1045 cm^−1^, which corresponds to the mixed lead–alkali glass composition [17,43], according to the composition measurements (Table 1). Cassiterite, which is characterized by a narrow peak at 633 cm^−1^ and a smaller one at 775 cm^−1^ [17,43,51], is only clearly detected in the MP (Figure 9a) and MF (Figure 9c) shards, at a very low intensity. The spectra characteristic of black spinel (MO, Figure 9a) are also detected, according to the detection of iron, manganese, and chromium, using XRF or SEM–EDS, in the paste. A strong doublet is observed at 1115 and 1300 cm^−1^ in the MB and MFF samples (Figure 9c). This feature is similar to the luminescence observed in the calcium-rich glass/glaze, especially those involving the nucleation of wollastonite (CaSiO_3_) [52,53].

#### 3.4.2. Raman Signatures of the Glaze and Painted Enamel Matrices

Figure 10 and Figure 11 compare selected Raman spectra recorded from the surface of the glazed background and the painted decorations.

The 635–775 cm^−1^ doublet of cassiterite is observed in all the shards except the MP and MPP İznik shards (Figure 10), despite EDS detecting tin concentrations between 0.14 and 0.29 at % in the MPP shard’s blue glaze (Table A2, Appendix A). This suggests that tin can remain dissolved during the glassy silicate phase, consistent with its incorporation as a lead impurity. Previous studies on early İznik production have shown that tin can be dissolved in the glaze at levels up to approximately 5–7 wt % SnO_2_ [42,54].

The Raman spectrum of a glassy silicate provides insight into the degree of polymerization of the SiO_4_ tetrahedron, and the structural and vibrational unit of silicates, whether crystalline or amorphous [50]. The center of gravity of the SiO_4_ stretching band ranges from ~950 (e.g., red areas in the MO shard, Figure 10b) to 1020 cm^−1^ (e.g., green areas in the MO shard, Figure 10b), corresponding to a lead-rich glass at the lower energy end and mixed lead–alkali glass at the higher energy end [17,50]. The polymerization index (I_p_), derived from the ratio of the bending to stretching modes of SiO_4_ [50], ranges from 0.3 (red) to 0.5 (green). These values correspond to glazes with varying melting behaviors and, consequently, different firing temperatures [50], typically between approximately ~600 and ~800 °C. For most colors, these values align with those observed in a prior study of a bowl [17] depicting *Faridun astride the cow*, *Birmaya*. However, in that bowl, the background glaze was nearly free of lead, with the primary stretching mode at 1100 cm^−1^. The İznik glaze Raman signature of the stretching band (Figure 10e,f) shows the characteristic 985–1040 cm^−1^ doublet [17,43].

#### 3.4.3. Raman Signatures of the Pigments

The peak observed between 815 and 830 cm^−1^ (Figure 10a,c, Table 2), characteristic of lead–potassium–calcium–sodium arsenates (various phases are possible, with their signatures being rather similar [55]), appears in the blue regions and is consistent with the use of Kashan cobalt ores. In many areas (Figure 10c), the AsO_4_ ion signal is absent. Since this signature is not detected throughout all the points of the blue zones, it is likely that the blue color arises from the ‘simple’ dissolution of Co^2+^ ions in the silicate glass network, accompanied by some precipitation of the arsenic-rich phase(s). Destructive Transmission Electron Microscopy or Synchrotron-µ X-ray diffraction will be required to gain a comprehensive understanding of the different phases and their distribution in the glazed layer.

The spectra obtained from the dark blue-colored regions, using blue laser excitation, reveal an additional intense peak around 12,501,350 cm^−1^ (Figure 10c), typical of enamels where borax is expected to have been added [56]. This peak corresponds to B-O stretching modes, which appear in this spectral range [56,57,58,59,60]. Matin and Pollard identified the mineral, ulexite (NaCaB_5_O_9_·8(H_2_O)), associated with Kashan cobalt ores [40,41]. Borax acts as a highly effective flux, enabling low-temperature fusions and reducing viscosity. Its use is well-documented in the preparation of blue-painted enamels, in the 18th century, on both soft and hard porcelain, in Europe and China [56,61,62]. Furthermore, borax was widely used in the decoration of İznik wares (16th century) [63] and in the preparation of certain Chinese glass objects (Liao Dynasty, 10th-12th centuries) [64]. The use of borax in the production of blue-painted enamels, although undoubtedly unintentional as it originates from a cobalt source, represents an important discovery. The dual association of arsenic and borax in Kashan cobalt ore is particularly advantageous for enameling.

The Raman spectra of black regions reveal the signature of chromate ions at 840 cm^−1^ (Figure 10d,e) [17,38,43]. In other cases, chromate is not detected, but a typical spinel signature (main peak at ~670 cm^−1^, Figure 10d,g) is observed, corresponding to a phase based on Mn and Fe [38,65].

Red areas exhibit the signature of hematite, with a series of peaks at ~200, 280, and 390 cm^−1^ (Figure 10b and Figure 11a), among others [66].

For turquoise and green colors, only glass spectra are observed, with the enamel colored by Cu^2+^ ions, without the formation of a crystalline phase (Figure 10g).

Table 3 lists the crystal phases identified.

Some of the enamels exhibit a peak around 950 cm⁻¹ (M, MPP, MF, Figure 10b,h and Figure 11a), which is too sharp to correspond to the SiO_4_ stretching mode of the glassy phase, and is more likely indicative of a phosphate [48,49], as the SEM–EDS measurements reveal a strong phosphorus signal for these shards.

**Figure 12:**
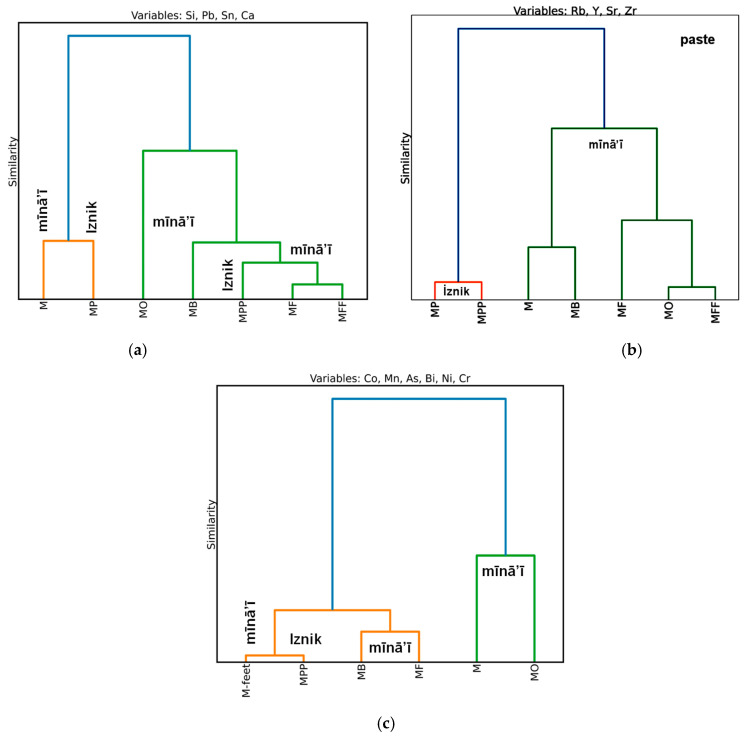
Hierarchical clustering classification of mīnā’ī and İznik artifacts based on: (**a**) major element content (Si, Pb, Sn, Ca) in the paste; (**b**) Rb, Y, Sr, and Zr impurities in the paste; and (**c**) elements associated with cobalt (Co, Mn, As, Bi, Ni, Cr) in the blue decoration.

As observed when comparing the ternary diagrams for the major elements, the similarities between the pastes in the mīnā’ī and İznik shards make hierarchical classification ineffective (Figure 12a) when using these variables. However, the use of impurities that give off a significant signal in terms of the XRF is effective (Figure 12b). This indicates the use of certain specific raw materials by each group.

**Figure 13:**
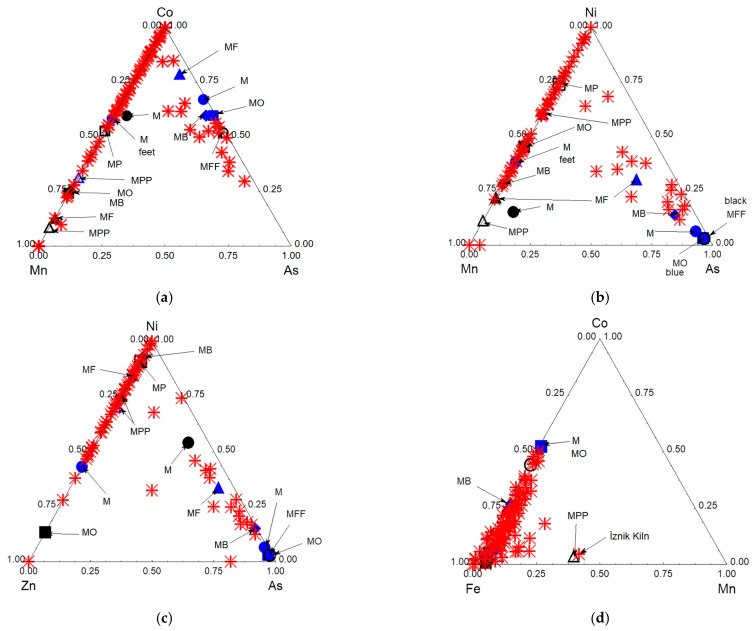
Comparison of the relative content of elements associated with cobalt (Mn, As, Ni, Zn and Fe) (**a**–**d**) in the blue (blue label) and dark blue to black (black label) glaze of the mīnā’ī shards (solid circle, triangle and square) and Edirne and İznik tiles (red stars). A minor subgroup of İznik tiles, excavated from the İznik tile kilns site, clusters near the Co–As vertex, while other shards from the İznik kilns and Edirne tiles [33,38,67,68], sourced from various mosques, are positioned near the Co–Mn vertex.

Classification based on cobalt and its associated elements (Figure 12c) imperfectly categorizes the mīnā’ī shards when all the blue-colored areas are considered, but does so perfectly when only the decorative areas are considered. Figure 13, which compares the cobalt signals and associated elements obtained for the mīnā’ī decorations with all the data collected from the various İznik productions, mainly tiles [33,67,68], shows that the light blue and dark blue areas belong to the same groups as those for the İznik decorations. These groups are characterized either by an association with manganese, in addition to nickel and zinc, with arsenic, while iron is always present. Thus, there are two types of cobalt sources (associated with Mn and As, respectively). The exclusive use of cobalt associated with arsenic for light blue areas indicates that Iranian potters understood the benefit of arsenic as an opacifier, enhancing the blue color.

## 4. Conclusions

Tin and lead were unexpectedly identified in the glassy phase (frit), cementing the mīnā’ī stonepaste body, using pXRF analysis. This finding was further confirmed using SEM–EDS analysis. Three distinct compositions of mīnā’ī paste were observed: first in the M shard; and then in the MF, MFF, and MB shards in regard to the second group; and, thirdly, in the MO shard. Each composition exhibited different impurities, suggesting two separate sites or periods of production for the studied samples.

The ability of pXRF to efficiently distinguish between different types of mīnā’ī paste compositions (and their associated impurities) holds great promise for the non-invasive identification of objects originating from various production locations, workshops, cities, or eras. By analyzing both the major or minor elements in the pastes (Si, Pb, Sn, Ca) and the associated impurities (Rb, Y, Sr, Zr), Ward’s hierarchical classification was able to effectively identify distinct groups. A relationship was observed with later İznik fritware bodies, although the classification remains inconclusive.

Regarding the blue-colored decoration, which is characteristic of Islamic production, classification using the pXRF signal of cobalt and associated elements (Mn, As, Bi, Ni, Cr) was very effective at differentiating the M and MO mīnā’ī shards from the others. This finding emphasizes the importance of conducting a series of pXRF measurements on a much larger sample corpus. Different types of black pigments were identified, including chromite, spinel, and cobalt-containing phases.

The Raman analyses, using a blue laser, indicate for the first time the presence of boron in blue-painted enamel, originating from the specific composition of Kashan cobalt ores.

The thinness of the painted enamel decoration makes Raman microspectroscopy, which is capable of analyzing very small volumes, one of the most effective techniques for characterizing the technical features of these decorations. Variations in the polymerization index and the wavenumber of the center of gravity of the SiO_4_ stretching mode indicate that painted enamels with different colors have distinct compositions, with varying PbO and B_2_O_3_ content. These differences require different firing temperatures and, consequently, multiple firing cycles. This complex firing *chaîne opératoire* is undoubtedly at the origin of the exceptional novelty of the enameled decoration found on mīnā’ī ware.

This innovative technology was later adopted by İznik potters, two centuries after its initial development. However, the precise relationship between Seljuk and Ottoman potters remains an open question.

## Figures and Tables

**Table 1:** Local compositions (at %) measured by SEM–EDS analysis for different spots on the body fracture at 400× magnification (~300 × 250 µm^2^ area). Full data are provided in Appendix A (Σ = Na + K + Ca).

Shard	Type	Si	Al	Si/Al	Mg	Σ	Pb	Sn	Pb/Sn	P	Fe	Ti	Co	Cr
MPP1	*İznik*	19.29	2.85	6.77	0.31	6.29	3.22	0.31	10.4	0.43	0.02	-	-	-
MPP2+	*İznik*	9.26	4.38	2.11	0.62	4	2.13	0.04	53.2	4.26	0.03	0.03	-	-
MPP3	*İznik*	31.98	1.65	19.38	0.24	3.64	0.23	-	-	0.43	0.15	-	-	-
MP1 ^a^	*İznik*	37.8	1.21	31.24	0.38	2.89	0.23	-	-	0.4	0.55	0.05	-	0.15
MP2	*İznik*	35.77	1.21	29.56	0.31	3.03	0.54	0.05	10.8	0.72	0.21	0.03	-	-
MO1 ^a^	mīnā’ī	25.04	3.49	7.17	1.33	6.09	1.85	0.38	4.86	0.17	0.53	0.01	0.19	0.13
MO2	mīnā’ī	20.57	4.6	4.47	1.04	4.22	1.15	0.13	8.84	0.10	0.02	0.02	-	-
MFF1	mīnā’ī	25.58	3.61	7.09	1.68	8.45	2.6	1.23	2.1	0.15	0.15	0.02	-	-
MFF2	mīnā’ī	28.09	5.69	4.94	0.81	6.48	-	-	-	0.15	0.58	0.22		0.08
MFF3 ^a^	mīnā’ī	27.06	5.54	4.88	1.24	5.24	1.89	0.39	4.85	0.14	0.23	0.03	0.14	-
MF1	mīnā’ī	25.52	4.34	5.88	0.55	5.1	-	-	-	0.14	0.21	0.23	-	-
MF2	mīnā’ī	18.37	3.61	5.09	0.64	5.29	1.17	1.89	0.61	0.65	0.29	0.04		

Notes: + magnification represents 2000×; ^a^ means close to the glaze.

**Table 2:** Characteristic Raman peak wavenumbers used to identify pigments and opacifiers and the main components of the SiO_4_ stretching modes.

Samples		Cream	White	Dark Blue	Blue	Light Blue	Red	Brown	Green	Turquoise	Black
Dimension (cm)	M										
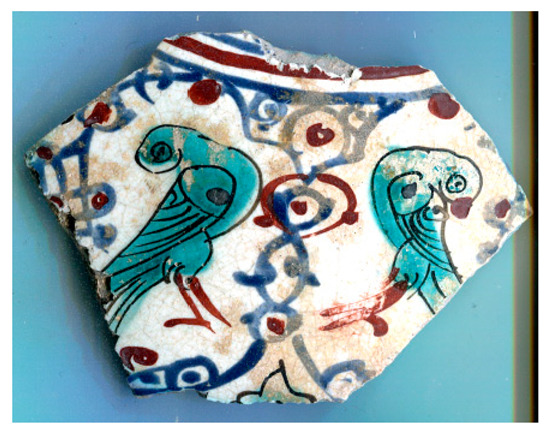 7 × 5.5 × 0.45	MO	150 513 685 960 1007 1115		832 995 1330	835 995 1330	633–775 832 990 1330					542 675 832 970
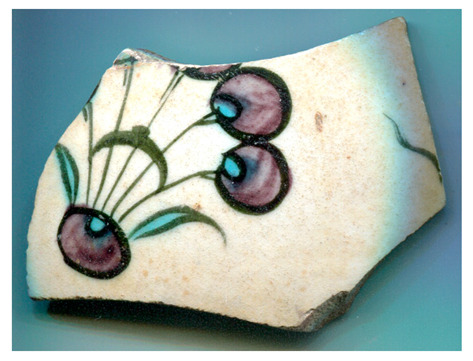 7 × 4 × 0.3	MP (İznik)							985 1045	985 1045		840 975 1030
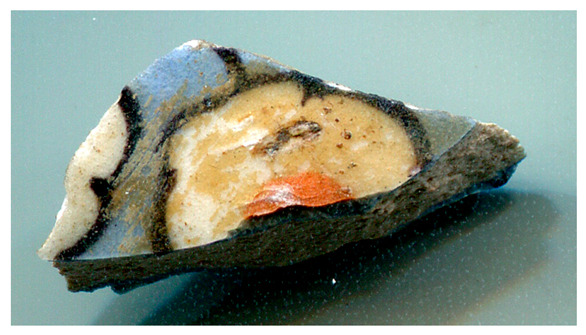 2 × 1 × 0.55	MPP (İznik)				985 1045				985 1045		
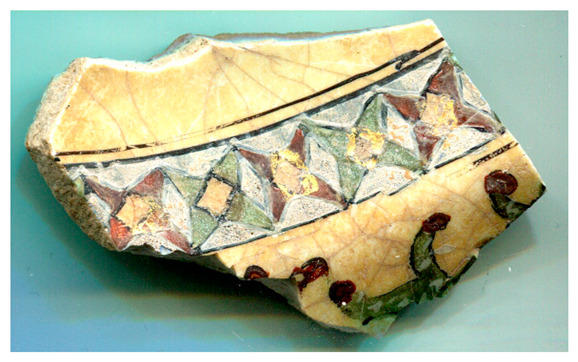 5 × 4 × 0.5	MF	633–775 985	613 830 675				200 280 535 950 1010		545 970 1008		550 685 955
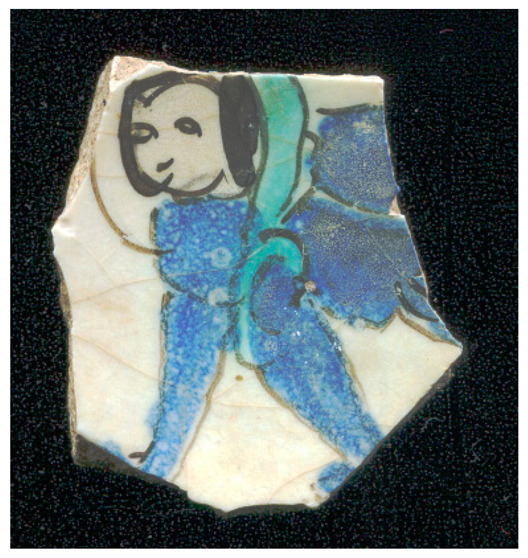 4 × 3 × 0.4	MB				633–775 820 9555 1120					475 985 1120	500 655 950
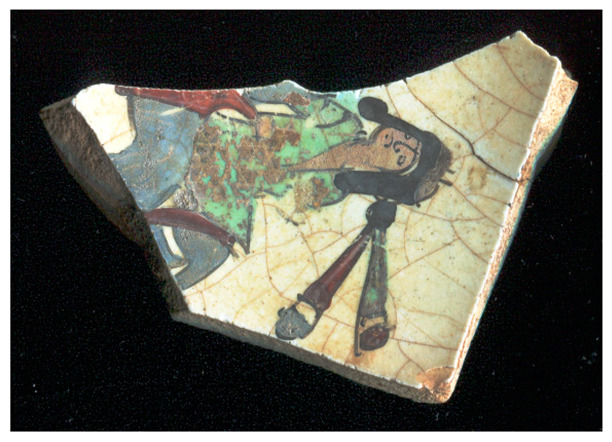 7.5 × 5 × 0.5	M										

**Table 3:** Pigments and opacifiers.

Shard	MO	MP (İznik)	MF	MPP (İznik)	MB	M	Bowl [17]
White	cassiterite		anatase? arsenate		cassiterite arsenate	-	
Cream			cassiterite	anatase	cassiterite	cassiterite	
Blue	arsenate			phosphate	cassiterite	arsenate	
Turquoise					no	cassiterite	cassiterite
Red/brown	hematite	-	hematite		-	hematite	
Orange		-					
Green		anatase	cassiterite			-	cassiterite
Black		chromite	spinel	chromite	spinel	spinel	

## Data Availability

The original contributions presented in this study are included in the article and Supplementary Materials. Further inquiries can be directed to the corresponding author

## Supplementary Materials

The following supporting information can be downloaded at: https://www.mdpi.com/article/10.3390/ma18030575/s1, Table S1: pXRF data.

## Appendix A

**Table A1:** Local compositions (at %) measured by SEM–EDS for different spots on the body fracture, with 400× magnification (~300 × 250 µm^2^ area).

Shard	O	Si	Al	Mg	Na	K	Ca	P	S	Cl	Pb	Sn	Pb/Sn	Fe	Ti	Co	Cr
MPP1	66.3	19.29	2.85	0.31	5.48	0.52	0.29	0.43	0.4	0.56	3.22	0.31	10.4	0.02	-	-	-
MPP2 +	73.75	9.26	4.38	0.62	2.42	0.36	1.22	4.26	0.98	0.52	2.13	0.04	53.2	0.03	0.03	-	-
MPP3	61.52	31.98	1.65	0.24	2.38	0.49	0.77	0.43	0.17		0.23	-	-	0.15	-	-	-
MP1 ^a^	55.77	37.8	1.21	0.38	1.49	0.41	0.99	0.4	0.18	0.4	0.23	-	-	0.55	0.05	-	0.15
MP2	57.64	35.77	1.21	0.31	1.42	0.35	1.26	0.72	0.19	0.31	0.54	0.05	10.8	0.21	0.03	-	-
MO1 ^a^	60.41	25.04	3.49	1.33	4.16	1.03	0.9	0.17	0.13	0.25	1.85	0.38	4.86	0.53	0.01	0.19	0.13
MO2	67.87	20.57	4.6	1.04	3.33	0.54	0.35	0.10	0.12	0.15	1.15	0.13	8.84	0.02	0.02	-	-
MFF1	55.82	25.58	3.61	1.68	6.04	1.11	1.30	0.15	0.18	0.52	2.6	1.23	2.1	0.15	0.02	-	-
MFF2	57.66	28.09	5.69	0.81	4.35	0.95	1.18	0.15	0.14	0.10	-	-	-	0.58	0.22		0.08
MFF3 ^a^	57.69	27.06	5.54	1.24	3.12	1.26	0.86	0.14	0.15	0.28	1.89	0.39	4.85	0.23	0.03	0.14	-
MF1	63.68	25.52	4.34	0.55	3.74	0.95	0.41	0.14	0.08	0.16	-	-	-	0.21	0.23	-	-
MF2	67.56	18.37	3.61	0.64	2.81	1.16	1.32	0.65	0.23	0.26	1.17	1.89	0.61	0.29	0.04		

Notes: + magnification means 2000×; ^a^ means close to the glaze.

**Table A2:** Local compositions (at %) measured by SEM–EDS at different spots in the MPP shard decoration.

Shard	Color	O	Si	Al	Mg	Na	K	Ca	P	S	Cl	Pb	Sn	Pb/Sn	Fe	Ti	Cu	Co
MPP	Blue1 ^a^	68.93	17.38	3.44	0.4	4.86	0.36	0.37	0.69	0.41	0.47	2.37	0.29	8.17	0.04	0.01	0.01	-
	Blue2 ^b^	72.04	13.59	7.27	0.59	3.66	0.18	0.06	0.18	0.30	0.31	1.5	0.2	7.5	0.02	-	0.02	-
	Blue3 ^b^	69.99	12.99	9.86	0.75	3.64	0.33	0.14	0.25	0.33	0.35	1.34	0.14	9.57	0.01	-	0.06	-
	Black1 ^a^	70.75	14.27	5.84	0.38	2.1	0.41	1.03	2.41	0.69	0.46	1.41	0.08	17.62	0.07	0.04	0.02	0.03
	Black2 ^b^	71.03	15.35	7.72	0.58	2.91	0.51	0.28	0.45	0.62	0.22	0.23	0.02	11.5	0.04	0.01	0.02	

Notes: ^a^ means magnification 150×; ^b^ means magnification 400×.
